# Transmissibility, hospitalization, and intensive care admissions due to omicron compared to delta variants of SARS-CoV-2 in Catalonia: A cohort study and ecological analysis

**DOI:** 10.3389/fpubh.2022.961030

**Published:** 2022-08-12

**Authors:** Martí Català, Ermengol Coma, Sergio Alonso, Cristina Andrés, Ignacio Blanco, Andrés Antón, Antoni E. Bordoy, Pere-Joan Cardona, Francesc Fina, Elisa Martró, Manuel Medina, Núria Mora, Verónica Saludes, Clara Prats, Daniel Prieto-Alhambra, Enrique Alvarez-Lacalle

**Affiliations:** ^1^Nuffield Department of Orthopedics, Rheumatology and Musculoskeletal Sciences (NDORMS), University of Oxford, Oxford, United Kingdom; ^2^Primary Care Services Information System (SISAP), Institut Català de la Salut (ICS), Barcelona, Spain; ^3^Physics Department, Universitat Politècnica de Catalunya, Barcelona, Spain; ^4^Respiratory Viruses Unit, Virology Section, Microbiology Department, Vall d'Hebron Hospital Universitari, Vall d'Hebron Institut de Recerca (VHIR), Vall d'Hebron Barcelona Hospital Campus, Barcelona, Spain; ^5^Biomedical Research Networking Center in Infectious Diseases (CIBERINF), Instituto de Salud Carlos III, Madrid, Spain; ^6^Clinical Genetics Department, Laboratori Clínic Metropolitana Nord, Hospital Universitari Germans Trias i Pujol, Institut d'Investigació en Ciències de la Salut Germans Trias i Pujol (IGTP), Badalona, Spain; ^7^Microbiology Department, Laboratori Clínic Metropolitana Nord, Hospital Universitari Germans Trias i Pujol, Institut d'Investigació en Ciències de la Salut Germans Trias i Pujol (IGTP), Badalona, Spain; ^8^Biomedical Research Networking Center in Respiratory Diseases (CIBERES), Instituto de Salud Carlos III, Madrid, Spain; ^9^Department of Genetics and Microbiology, Universitat Autònoma de Barcelona, Cerdanyola, Spain; ^10^Biomedical Research Networking Center in Epidemiology and Public Health (CIBERESP), Instituto de Salud Carlos III, Madrid, Spain

**Keywords:** COVID-19, severity, ecological study, cohorts, substitution model, severity and vaccination status

## Abstract

**Purpose:**

We aim to compare the severity of infections between omicron and delta variants in 609,352 SARS-CoV-2 positive cases using local hospitalization, vaccination, and variants data from the Catalan Health Care System (which covers around 7. 8 million people).

**Methods:**

We performed a substitution model to establish the increase in transmissibility of omicron using variant screening data from primary care practices (PCP) and hospital admissions. In addition, we used this data from PCP to establish the two periods when delta and omicron were, respectively, dominant (above 95% of cases). After that, we performed a population-based cohort analysis to calculate the rates of hospital and intensive care unit (ICU) admissions for both periods and to estimate reduction in severity. Rate ratios (RR) and 95% confidence intervals (95% CI) were calculated and stratified by age and vaccination status. In a second analysis, the differential substitution model in primary care vs. hospitals allowed us to obtain a population-level average change in severity.

**Results:**

We have included 48,874 cases during the delta period and 560,658 during the omicron period. During the delta period, on average, 3.8% of the detected cases required hospitalization for COVID-19. This percentage dropped to 0.9% with omicron [RR of 0.46 (95% CI: 0.43 to 0.49)]. For ICU admissions, it dropped from 0.8 to 0.1% [RR 0.25 (95% CI: 0.21 to 0.28)]. The proportion of cases hospitalized or admitted to ICU was lower in the vaccinated groups, independently of the variant. Omicron was associated with a reduction in risk of admission to hospital and ICU in all age and vaccination status strata. The differential substitution models showed an average RR between 0.19 and 0.50.

**Conclusion:**

Both independent methods consistently show an important decrease in severity for omicron relative to delta. The systematic reduction happens regardless of age. The severity is also reduced for non-vaccinated and vaccinated groups, but it remains always higher in the non-vaccinated population. This suggests an overall reduction in severity, which could be intrinsic to the omicron variant. The fact is that the RR in ICU admission is systematically smaller than in hospitalization points in the same direction.

## Introduction

On 26 November 2021, the World Health Organization (WHO) declared omicron (Pango lineage B.1.1.529) a SARS-CoV-2 variant of concern (VOC) (1). Infections with omicron increased rapidly in Europe and became the dominant variant within a few weeks. Omicron has been shown to be highly transmissible, with a capacity of infection and reinfection between two and three times higher than the delta variant (2). This led to a rapid substitution not only in Europe but also in the United States and different Asian countries.

The appearance of the omicron variant has generated an important debate about a possible paradigm shift in the way of dealing with the pandemic. Different data have suggested that the omicron variant is less severe than the delta variant (3–5). More specifically, smaller ratios of hospitalization and intensive care unit (ICU) admission per case detected have been systematically found even when corrected for age, sex, or vaccination status (3). These lower ratios imply that the same healthcare resources can face a much higher circulation of the virus. This would allow a reduction or even elimination of non-pharmacological interventions (NPIs), such as those carried out by European countries (i.e., Denmark, UK), while keeping COVID-19 transmission within manageable levels. While not being the endgame of the pandemic, its future dynamics would be more directly related with the time-lapse of waning immunity and seasonal effects. Similarly, the comparison of hospitalization ratios at different times could lead to misleading conclusions due to environmental effects related to intrinsic immunity, timing of vaccination coverage, and intensity of NPIs. For example, there are indications of less mucosal immunity protection with colder and drier air (6), which might lead to different hospitalization rates just because of such environmental conditions. To minimize the effect of these changes, comparison of hospitalization rates should be carried out as continuously as possible, checking that any possible change due to a new variant is consistent across the substitution process.

The aim of our analysis was to leverage the large case count and hospitalization database of the Catalan Health System to compare the severity of infection between omicron and delta variants using local hospitalization, vaccination, and variants data. We used this information to quantify the increase in transmissibility due to omicron and to estimate derived hospitalization and ICU ratios stratified by age and vaccination status. Furthermore, we validated these results using a variant substitution model among hospitalizations over time. Finally, we addressed whether the observed decrease in severity offsets the increase in transmissibility associated with omicron.

## Methods

The study comprises the SARS-CoV-2 epidemic period from 01 November 2021 to 25 January 2022 in Catalonia, a region with 7.8 M in the northeast of Spain. This period corresponds to the expansion phase of the sixth wave, during which the delta variant was substituted by the omicron one. A substitution model was fitted to establish the increase in transmissibility of omicron using variant screening data from primary care settings in Catalonia, generated within the SARS-CoV-2 genomic surveillance program of the Catalan Health System. We also performed two distinct analyses of the severity of the omicron variant. We first used variant data from primary care centers to delimit the two periods when delta and omicron were, respectively, dominant (above 95% of cases) and to estimate both hospitalization and ICU ratios for each period. Second, a differential substitution model was fitted jointly to variants data in primary care and hospitals to analyze changes in severity by assessing the differential substitution properties in primary care vs. hospitals, inferring an average decrease in severity.

### Variant identification

In order to determine the different periods, the percentage of omicron presence was obtained through variants analyses carried out in the clinical microbiology laboratories at Hospital Universitari Vall d'Hebron (Barcelona, Catalonia, Spain) and Hospital Universitari Germans Trias i Pujol (Badalona, Catalonia, Spain) using a 20% of SARS-CoV-2-positive specimens from the primary care of their area of influence. From epidemiological weeks 48/2021 (late November) to 04/2022 (late January), screening of presumptive variants with ΔH69-ΔV70 in spike protein was first performed using the TaqPathTM COVID-19 RT-PCR Kit (Thermo Fisher Scientific, USA) according to the manufacturer's instructions. ΔH69-ΔV70 viruses are presumably detected with the assay when both ORF1ab and N targets yield positive amplifications with PCR cycles below 30, while the S target provides negative results due to the serendipitous location of its probes, known as S gene target failure (SGTF) (7). Those samples without SGTF were considered to be delta. These results were further confirmed using whole-genome sequencing techniques, analyzing a representative subset of the samples. The sensitivity and specificity of the PCR test were >99%, guaranteeing the accuracy of the omicron/delta ratios.

For the analysis of the substitution process in hospitals, the same variant analysis was performed but took into account the samples of all admitted patients in the two hospitals with SARS-CoV-2-positive specimens.

### Substitution model in transmission at the population level

We modeled the substitution of a variant A by a variant B as two independent epidemics that share all characteristics except for the transmissibility, with variant B being more transmissible than A. The model was used to estimate the daily percentage of cases that corresponded to each of the variants after being fitted to weekly SGTF screening determinations. Then, we calculated the effective reproduction number (R) that corresponds to each subset of cases, using an empiric definition (8).

The number of cases of each variant, *N*_*A*_ and *N*_*B*_, at a given moment, t, would evolve as follows:


$$\begin{array}{l} N_{A}=N_{A,0}e^{\beta_{A}t} \end{array}$$

$$\begin{array}{l} N_{B}=N_{B,0}e^{\beta_{B}t} \end{array}$$

where β_*A*_ and β_*B*_ are two exponents related with the transmissibility of each variant in this context and, therefore, to the effective reproduction number. The effective reproduction number, R, of each variant was assessed from these Equations, assuming a fixed mean period τ between infection and maximum infectivity of individuals (9):


$$\begin{array}{l} R_{A}\left(t\right)=\frac{N_{A}\left(t+\tau \right)}{N_{A}\left(t\right)}=e^{\beta_{A}\;\tau } \end{array}$$

$$\begin{array}{l} R_{B}\left(t\right)=\frac{N_{B}\left(t+\tau \right)}{N_{B}\left(t\right)}=e^{\beta_{B}\;\tau } \end{array}$$

Therefore, we could determine the values of the exponents from the effective reproduction numbers as $\beta_{A}=\frac{\ln\;\left(R_{A}\right)\;}{\tau }$ and $\beta_{B}=\frac{\ln\;\left(R_{B}\right)\;}{\tau }$. If variant B presents a population-level increase in transmissibility of η with respect to variant A, that is, *R*_*B*_ = η*R*_*A*_, the exponents will be related as $\beta_{B}=\frac{\ln\;\left(\eta R_{A}\right)\;}{\tau }=\text{Δ}\beta +\;\beta_{A}$ where $\text{Δ}\beta =\frac{\ln\;\left(\eta \right)\;}{\tau }$. Therefore, we calculated the increase in transmissibility η = *e*^Δ*βτ*^. In case the period τ depends on the variant, the transmissibility is $\eta =e^{\text{Δ}\beta \tau_{B}}\;e^{\beta_{A}\text{ Δ}\tau }$, where τ_*B*_ and τ_*A*_ are the corresponding periods and Δτ is the difference between them.

Given a certain initial ratio between cases of variant B and cases of variant A, ξ_0_ = *N*_*B*, 0_ /*N*_*A*, 0_, we modeled the fraction ρ_*B*_ of variant B with time as follows:


$$\begin{array}{l} \rho_{B}\left(t\right)=\frac{N_{B}\left(t\right)}{N_{A}\left(t\right)+N_{B}\left(t\right)}=\frac{N_{B,0}\;e^{\beta_{B}\;t}}{N_{A,0}e^{\beta_{A}t}+N_{B,0}e^{\beta_{B}t}}=\frac{\xi_{0}e^{\text{Δ}\beta t}}{1+\xi_{0}\;e^{\text{Δ}\beta t}} \end{array}$$

From the fit of this function, we estimate the daily percentage of cases that corresponded to each of the variants after being fitted to weekly sequencing determinations. Then, we calculated the effective reproduction number that corresponds to each subset of cases, using an empiric definition.

If the period between new cases is the same (τ_*A*_ = τ_*B*_ = τ), the increase in transmissibility can be computed as follows:


$$\begin{array}{l} \eta =e^{\text{Δ}\beta \tau } \end{array}$$

This quantity does not depend on the effective reproduction number of any of the epidemics [*R*_*A*_(*t*), *R*_*B*_(*t*)], but if the mean period between new cases is not the same, there is a dependency on the effective reproductive number. To compute the increase of transmissibility in this case, we need to estimate the mean effective reproduction number of at least one of the epidemics during a time period ($\bar{R_{A}}$ or $\bar{R_{B}}$):


$$\begin{array}{l} \eta =e^{\text{Δ}\beta \tau_{B}}\cdot {\bar{R_{A}}}^{\text{Δ}\tau /\tau_{A}}=e^{\text{Δ}\beta \tau_{A}}\cdot {\bar{R_{B}}}^{\text{Δ}\tau /\tau_{B}}. \end{array}$$

### Omicron and delta severity analysis

We performed a population-based cohort analysis. Data were obtained from the regional central database of laboratory confirmations by using reverse transcriptase polymerase chain reaction (RT-PCR) and lateral flow tests (LFT) for SARS-CoV-2 linked to hospital and ICU admissions. Vaccination status was obtained from the Catalan Shared Clinical Records, a database with vaccine data covering the entire Catalan Health System and all its vaccination centers.

The study population comprised two cohorts older than 10 years of age and defined based on the period in which each variant caused more than 95% of the analyzed cases, according to the results of the substitution model (see Results Section): (1) the delta cohort with initial SARS-CoV-2 infection identified between 01 November 2021 and 08 December 2021, and (2) the omicron cohort of cases identified between 05 January 2021 and 25 January 2022.

The main outcome was hospitalization and ICU admission in the 14-day time window following SARS-CoV-2 infection.

For this analysis, we excluded those individuals with a previous SARS-CoV-2 infection before the beginning of the study period.

### Statistical analysis

We compared the percentage of cases hospitalized or admitted to ICU in the following 14 days between omicron vs. delta cohorts. Rate ratios (RR) and 95% confidence intervals (95% CI) were calculated and stratified by age and vaccination status: non-vaccinated, partially vaccinated (one dose of a two-dose regimen vaccine), fully vaccinated (the complete vaccination regimen), and boosted (third dose). In addition, Mantel–Haenszel method was used to estimate overall adjusted RRs.

### Substitution model in admissions

We checked whether the severity reduction calculated was consistent with the substitution process observed in admissions to hospitals during the transition from the delta period to the omicron period. We used the substitution model adapted to hospital admissions. The model provides an expression for the proportion of omicron cases in hospital admission ρ_*H*_(*t*) as a function of time and the average RR between hospitalization with omicron and delta α.

We assumed a delay (*T*) between case confirmations and hospital admission, and an omicron admission rate different from that for delta. Generally speaking, we defined *r* as the average ratio of cases that led to hospitalizations for variant A (*H*_*A*_) (delta), obtaining the number of hospitalizations as a function of time as


$$\begin{array}{l} H_{A}=r\;N_{A0}e^{\beta_{A}\left(t-T\right)} \end{array}$$

We considered the average RR between hospitalization with variant B (omicron) and variant A (delta) as α to obtain


$$\begin{array}{l} H_{B}=\alpha r\;N_{B0}e^{\beta_{B}\left(t-T\right)} \end{array}$$

The proportion of variant B cases in hospital admissions ρ_*H*_*B*__ could be written as follows:


$$\begin{array}{l} \rho_{H_{B}}\left(t\right)=\frac{H_{B}}{H_{A}+H_{B}}=\frac{\alpha \xi_{o}e^{\text{Δ}\beta \left(t-T\right)}}{1+\alpha \xi_{o}e^{\text{Δ}\beta \left(t-T\right)}}=\frac{\xi_{{}_{H,0}}e^{\text{Δ}\beta t}}{1+\xi_{{}_{H,0}}e^{\text{Δ}\beta t}} \end{array}$$

where ξ__*H*, 0__ is the initial ratio between hospitalization with omicron and delta variants. This parameter was adjusted from the data. An important relation for our purposes is that α can be written as a combination of the adjusted parameters Δβ, ξ_*o*_ ξ__*H*, 0__, and *T* as


$$\begin{array}{l} \alpha =\frac{\xi_{{}_{H,0}}}{\xi_{o}}\;e^{\text{Δ}\beta T} \end{array}$$

Parameters Δβ, ξ_*o*_, and ξ__*H*, 0__ were obtained from the fit of the substitution process. The delay (*T*) between detection and hospitalization was obtained using the database of detected cases and admission from the mean value of the difference between diagnosis and hospital admission of the individuals detected between 09 December 2021 and 04 January 2022 (*N* = 3,298 individuals). The few cases that were negative or longer than 14 days were omitted to guarantee that there was a causal link between infection, detection, and admission.

The fit of the parameters of this model (Δβ, ξ__*H*, 0__) to experimental data of the substitution process in hospitals allows to compute α, being $\alpha =\frac{\xi_{{}_{H,0}}}{\xi_{o}}\;e^{\text{Δ}\beta T}$ with ξ_*o*_ being the initial ratio between cases of omicron and delta, once the delay (*T*) between detection and hospitalization is obtained using the database of detected cases and admissions.

## Results

### Increase in transmission

**Figure 1:**
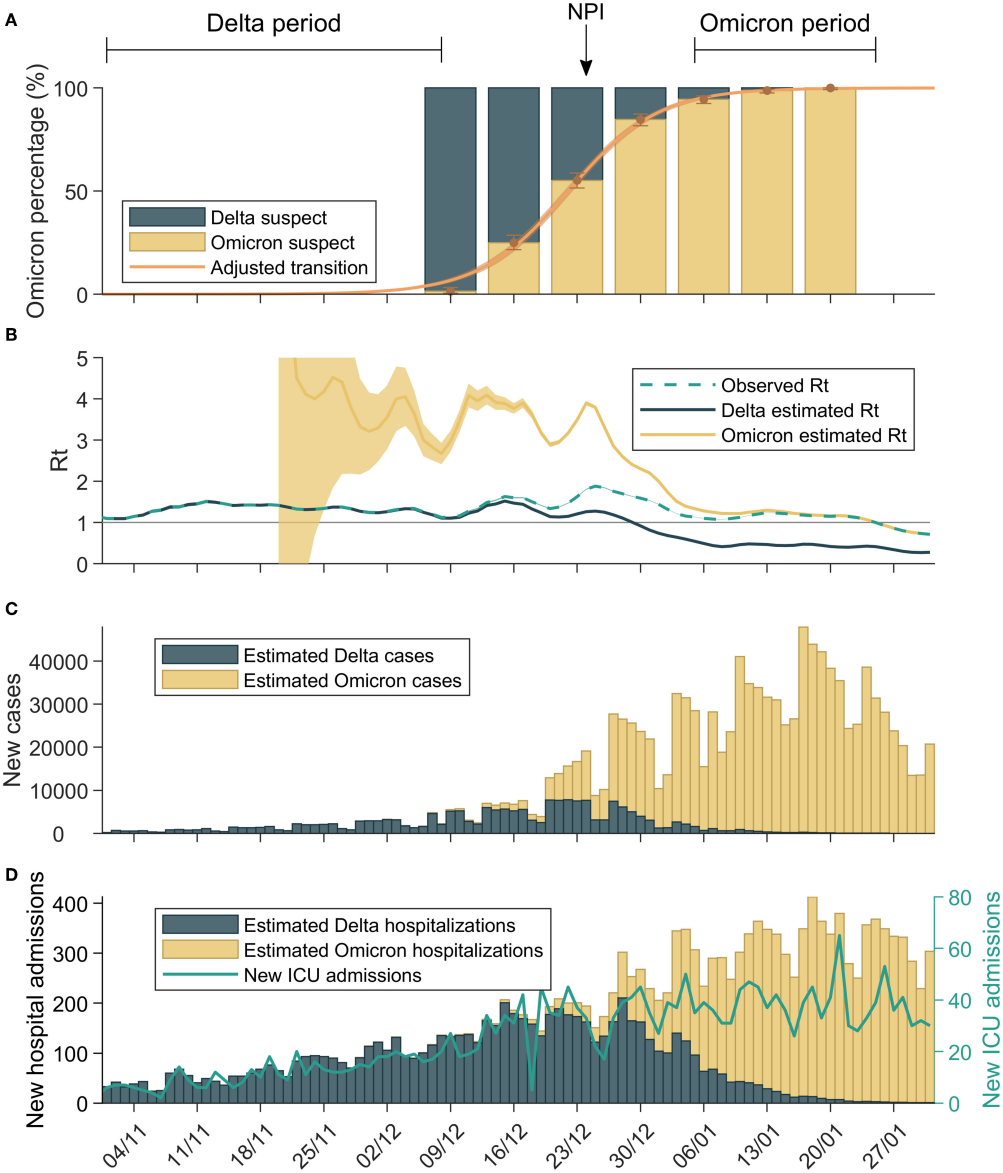
Omicron emergence in Catalonia. **(A)** Evolution of the omicron fitted percentage over time, using the substitution model, together with data from PCR screened samples. The two periods considered in the study are also indicated. **(B)** Empiric reproduction number of population-level incidence, together with the empiric reproduction number estimated for each variant. **(C)** Daily cases of each variant, estimated with the substitution model fitted to data. **(D)** Daily hospital admissions of each variant, estimated with the substitution model fitted to data of variants determinations among patients admitted to participant hospitals. The continuous line shows total admissions to intensive care units (variant data not available).

Primary care samples analyzed with PCR in participating sites of the Catalan SARS-CoV-2 Sequencing Network showed that omicron represented <3% of cases in epidemiological week 49/2021, increasing to >50% of the cases 2 weeks later (see Table 1). The substitution model was successfully fitted to weekly screening data of variants (r^2^ = 0.9968), as shown in (Figure 1A). The value of parameter Δβ resulting from the fitting was 0.209 [95% CI: 0.196, 0.222]. From this parameter, the increase in transmissibility η associated with omicron with respect to delta was computed depending on the generation time of each variant. The generation time was not the subject of our analysis. As a measure of the sensitivity to these parameters, we computed them under two scenarios. For a generation time of 5 days for both variants, η was 185% [95% CI: 166%−204%] while considering 4 days for the generation time of delta, and 3 days for the generation time of omicron led to an increase in transmissibility of 82% [95% CI: 72%−90%]. To compute these values, a mean R [1.14 (95% CI:1.0–1.3)] was used in the transition period (8).

**Table 1:** For each week, table shows the total number of cases for the whole Catalan system, the number of samples screened, and the number and rate of omicron detected.

**Week**	**Overall cases**	**N samples**	**Omicron samples**	**%**	**95% CI**
49/2021	17,742	349	5	1.4	[0.5–3.3]
50/2021	24,207	600	150	25.0	[21.6–28.7]
51/2021	41,682	739	408	55.2	[51.5–58.8]
52/2021	90,500	640	543	84.8	[81.8–87.5]
01/2022	145,349	653	617	94.5	[92.4–96.1]
02/2022	167,785	632	624	98.7	[97.5–99.5]
03/2022	220,146	425	425	100.0	[99.1–100.0]

Samples were collected from primary care cases in the hospital's area of influence and first analyzed with TaqPathTM COVID-19 RT-PCR Kit with SGTF as a source of rapid identification. Ratios were later confirmed with whole-genome sequencing techniques.

This increase in transmissibility is reflected in the R of each variant shown in (Figure 1B) where the delta variant had an Rt at 1–1.3 while omicron was at 3–4 during the last two weeks of 2021. Figure 1 also shows a systematic decrease in the R in Catalonia at the end week 51/2021.

Finally, we observed the effects of increased transmissibility and reduction in hospitalization and ICU rates in the total level of hospital admissions due to COVID-19. From week 50–51/2021, when delta had its peak of 7-day incidence at 0.6%, incidence increased up to the third week of 2022, where the peak of omicron was detected with a 7-day incidence of 3% (Figure 1C). Figure 1D shows that, as a consequence, the number of patients hospitalized increased with time together with the increase in omicron cases. On the contrary, the increase in transmission did not lead to an increase in ICU admission.

### Reduction in severity

**Figure 2:**
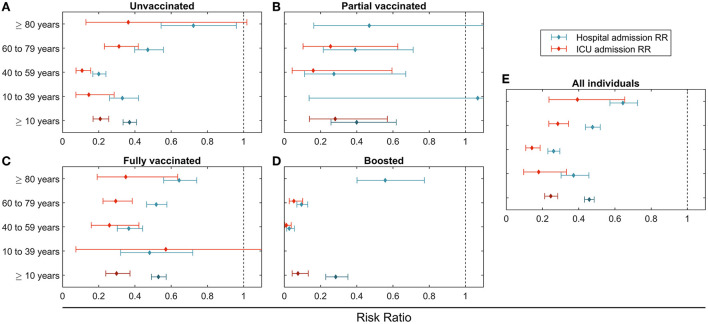
Rate ratio (RR) estimation between omicron and delta cohorts for the different vaccination status **(A–E)** and age groups (vertical axes). Hospital admission RR in blue and intensive care unit admission RR in red. **(A)** Unvaccinated; **(B)** Partial vaccinated; **(C)** Fully vaccinated; **(D)** Boosted; **(E)** All individuals.

We have analyzed 609,532 cases of which 560,658 (92%) were during the omicron period and 48,874 (8%) during the delta period. Cases analyzed during the delta period were 28.4% not vaccinated, 0.9% partially vaccinated, 69.4% fully vaccinated without booster, and 1.2% boosted. Cases detected during the omicron period were 18.0% not vaccinated, 0.7% partially vaccinated, 65.4% fully vaccinated without booster, and 16.0% boosted. The percentage of the detected cases that were admitted to hospitals and ICU is presented in Table 2 for both the delta and omicron periods for people older than 10 years. Table 3 and Figure 2 show the rate ratio (RR) associated with the data.

**Table 2:** Number of cases and hospitalizations and ICU admissions reported in each period, and percentage of cases with hospitalization and ICU admissions within 14 days.

	**Delta period (01/11/2021–08/12/2021)**							**Omicron period (05/01/2022–25/01/2022)**						
	**Case**	**Hosp**	**Prop**	**95% CI**	**ICU**	**Prop**	**95% CI**	**Case**	**Hosp**	**Prop**	**95% CI**	**ICU**	**Prop**	**95% CI**
**Global**
≥10	48,874	1,881	3.8%	[3.7–4.0]	384	0.8%	[0.7–0.9]	560,658	4,886	0.9%	[0.8–0.9]	505	0.1%	[0.1–0.1]
10 to 39	19,145	119	0.6%	[0.5–0.7]	17	0.1%	[0.1–0.1]	279,917	520	0.2%	[0.2–0.2]	32	0.0%	[0.0–0.0]
40 to 59	18,184	361	2.0%	[1.8–2.2]	99	0.5%	[0.4–0.7]	207,552	913	0.4%	[0.4–0.5]	130	0.1%	[0.1–0.1]
60 to 79	9,482	903	9.5%	[8.9–10.1]	231	2.4%	[2.1–2.8]	54,895	1,818	3.3%	[3.2–3.5]	291	0.5%	[0.5–0.6]
≥80	2,063	498	24.1%	[22.3–26.0]	37	1.8%	[1.3–2.5]	18,294	1,635	8.9%	[8.5–9.4]	52	0.3%	[0.2–0.4]
**Not vaccinated**
≥10	13,875	558	4.0%	[3.7–4.4]	164	1.2%	[1.0–1.4]	100,596	1,249	1.2%	[1.2–1.3]	189	0.2%	[0.2–0.2]
10 to 39	9,885	92	0.9%	[0.8–1.1]	16	0.2%	[0.1–0.3]	76,471	235	0.3%	[0.3–0.3]	18	0.0%	[0.0–0.0]
40 to 59	2,954	212	7.2%	[6.3–8.2]	70	2.4%	[1.9–3.0]	18,781	270	1.4%	[1.3–1.6]	48	0.3%	[0.2–0.3]
60 to 79	893	197	22.1%	[19.4–24.9]	73	8.2%	[6.5–10.2]	4,320	449	10.4%	[9.5–11.3]	110	2.5%	[2.1–3.1]
≥80	143	57	39.9%	[31.8–48.4]	5	3.5%	[1.1–8.0]	1,24	295	28.8%	[26.1–31.7]	13	1.3%	[0.7–2.2]
**Partially vaccinated**
≥10	457	30	6.6%	[4.5–9.2]	13	2.8%	[1.5–4.8]	3,717	65	1.7%	[1.4–2.2]	19	0.5%	[0.3–0.8]
10 to 39	247	1	0.4%	[0.0–2.2]	0	0.0%	[0.0–1.5]	2,314	10	0.4%	[0.2–0.8]	1	0.0%	[0.0–0.2]
40 to 59	139	7	5.0%	[2.0–10.1]	4	2.9%	[0.8–7.2]	1,89	15	1.4%	[0.8–2.3]	5	0.5%	[0.1–1.1]
60 to 79	58	17	29.3%	[18.1–42.7]	9	15.5%	[7.3–27.4]	253	29	11.5%	[7.8–16.0]	10	4.0%	[1.9–7.1]
≥80	13	5	38.5%	[13.9–68.4]	0	0.0%	[0.0–24.7]	61	11	18.0%	[9.4–30.0]	3	4.9%	[1.0–13.7]
**Fully vaccinated**
≥10	33,898	1,204	3.6%	[3.4–3.8]	194	0.6%	[0.5–0.7]	366,060	1,826	0.5%	[0.5–0.5]	171	0.0%	[0.0–0.1]
10 to 39	8,972	26	0.3%	[0.2–0.4]	1	0.0%	[0.0–0.1]	188,777	263	0.1%	[0.1–0.2]	12	0.0%	[0.0–0.0]
40 to 59	15,019	134	0.9%	[0.7–1.1]	22	0.1%	[0.1–0.2]	158,083	516	0.3%	[0.3–0.4]	60	0.0%	[0.0–0.0]
60 to 79	8,322	646	7.8%	[7.2–8.4]	139	1.7%	[1.4–2.0]	16,929	680	4.0%	[3.7–4.3]	83	0.5%	[0.4–0.6]
≥80	1,585	398	25.1%	[23.0–27.3]	32	2.0%	[1.4–2.8]	2,271	367	16.2%	[14.7–17.7]	16	0.7%	[0.4–1.1]
**Booster**
≥10	596	87	14.6%	[11.9–17.7]	13	2.2%	[1.2–3.7]	89,268	1,742	2.0%	[1.9–2.0]	125	0.1%	[0.1–0.2]
10 to 39	18	0	0.0%	[0.0–18.5]	0	0.0%	[0.0–18.5]	11,834	12	0.1%	[0.1–0.2]	1	0.0%	[0.0–0.0]
40 to 59	57	8	14.0%	[6.3–25.8]	3	5.3%	[1.1–14.6]	29,182	110	0.4%	[0.3–0.5]	17	0.1%	[0.0–0.1]
60 to 79	201	42	20.9%	[15.5–27.2]	10	5.0%	[2.4–9.0]	33,320	658	2.0%	[1.8–2.1]	87	0.3%	[0.2–0.3]
≥80	320	37	11.6%	[8.3–15.6]	0	0.0%	[0.0–1.1]	14,932	962	6.4%	[6.1–6.8]	20	0.1%	[0.1–0.2]

**Table 3:** Rate ratio (RR) estimation comparing omicron vs. delta periods stratified by vaccination status and age groups.

	**Hospital admissions**		**ICU admissions**	
	**RR**	**95% CI**	**RR**	**95% CI**
**Global**
≥10*	0.458	[0.432–0.485]	0.245	[0.212–0.284]
10 to 39*	0.371	[0.303–0.456]	0.179	[0.096–0.332]
40 to 59*	0.261	[0.230–0.296]	0.142	[0.107–0.186]
60 to 79*	0.476	[0.437–0.519]	0.284	[0.235–0.344]
≥80*	0.644	[0.572–0.725]	0.392	[0.235–0.654]
**Not vaccinated**
≥10*	0.370	[0.334–0.408]	0.208	[0.169–0.256]
10 to 39	0.330	[0.259–0.420]	0.145	[0.074–0.285]
40 to 59	0.200	[0.167–0.240]	0.108	[0.075–0.156]
60 to 79	0.471	[0.398–0.557]	0.311	[0.232–0.419]
≥80	0.723	[0.544–0.960]	0.363	[0.129–1.018]
**Partially vaccinated**
≥10*	0.399	[0.258–0.618]	0.281	[0.139–0.569]
10 to 39	1.067	[0.137–8.338]	—	—
40 to 59	0.274	[0.112–0.671]	0.160	[0.043–0.594]
60 to 79	0.391	[0.215–0.712]	0.255	[0.104–0.627]
≥80	0.469	[0.163–1.349]	—	—
**Fully vaccinated**
≥10*	0.530	[0.491–0.572]	0.298	[0.239–0.372]
10 to 39	0.481	[0.321–0.719]	0.570	[0.074–4.386]
40 to 59	0.366	[0.303–0.442]	0.259	[0.159–0.422]
60 to 79	0.517	[0.465–0.576]	0.294	[0.224–0.385]
≥80	0.644	[0.558–0.742]	0.349	[0.191–0.636]
**Booster**
≥10*	0.283	[0.229–0.351]	0.075	[0.043–0.132]
10 to 39	—	—	—	—
40 to 59	0.027	[0.013–0.055]	0.011	[0.003–0.038]
60 to 79	0.095	[0.069–0.129]	0.052	[0.027–0.101]
≥80	0.557	[0.401–0.774]	—	—

Mantel–Haenszel method was used to estimate overall pooled estimates (^*^).

On average, 3.8% of the detected cases required hospitalization for COVID-19 complications during the delta period. This percentage dropped to 0.9% with omicron, equivalent to an RR of 0.46 [95% CI: 0.43 to 0.49], as shown in Table 3. In addition, the percentage of cases with an ICU admission in the 14-day window dropped from 0.8% in the delta cohort to 0.1% in the omicron cohort [RR 0.25 (95% CI: 0.21 to 0.28)]. The resulting RR of hospitalization and ICU admission increased for cohorts older than 60 and decreased for younger ages. For example, for those aged 10 to 39, RR for hospitalization and ICU admission was 0.37 (95% CI: 0.30 to 0.46) and 0.18 (95% CI: 0.10 to 0.33), while for those older than 80, the equivalent RR was higher, 0.64 (95% CI: 0.57 to 0.72) and 0.39 (95% CI: 0.23 to 0.65), respectively.

The proportion of cases hospitalized or admitted to ICU was lower in the vaccinated groups, independently of the variant. Full vaccination without booster did not modify the observed reduction in severity for omicron relative to delta, as shown by stratification by vaccination status in Table 3. For example, for the age cohort from 60 to 79, the unvaccinated population had an RR for hospitalization of 0.47 [95% CI: 0.40 to 0.56], while for the fully vaccinated RR was 0.52 [95% CI: 0.46 to 0.58]. For the boosted population, age cohorts between 40 and 79 years old had smaller RR. In particular, the age cohorts from 60 to 79 had an RR for hospitalization of 0.10 [95% CI: 0.07 to 0.13]. The boosted population older than 80 did not present differences in RR for hospitalization from the fully vaccinated.

Omicron was associated with a reduction in risk of admission to hospital and ICU in all age and vaccination status strata.

### Reduction in severity using differential substitution model

**Figure 3:**
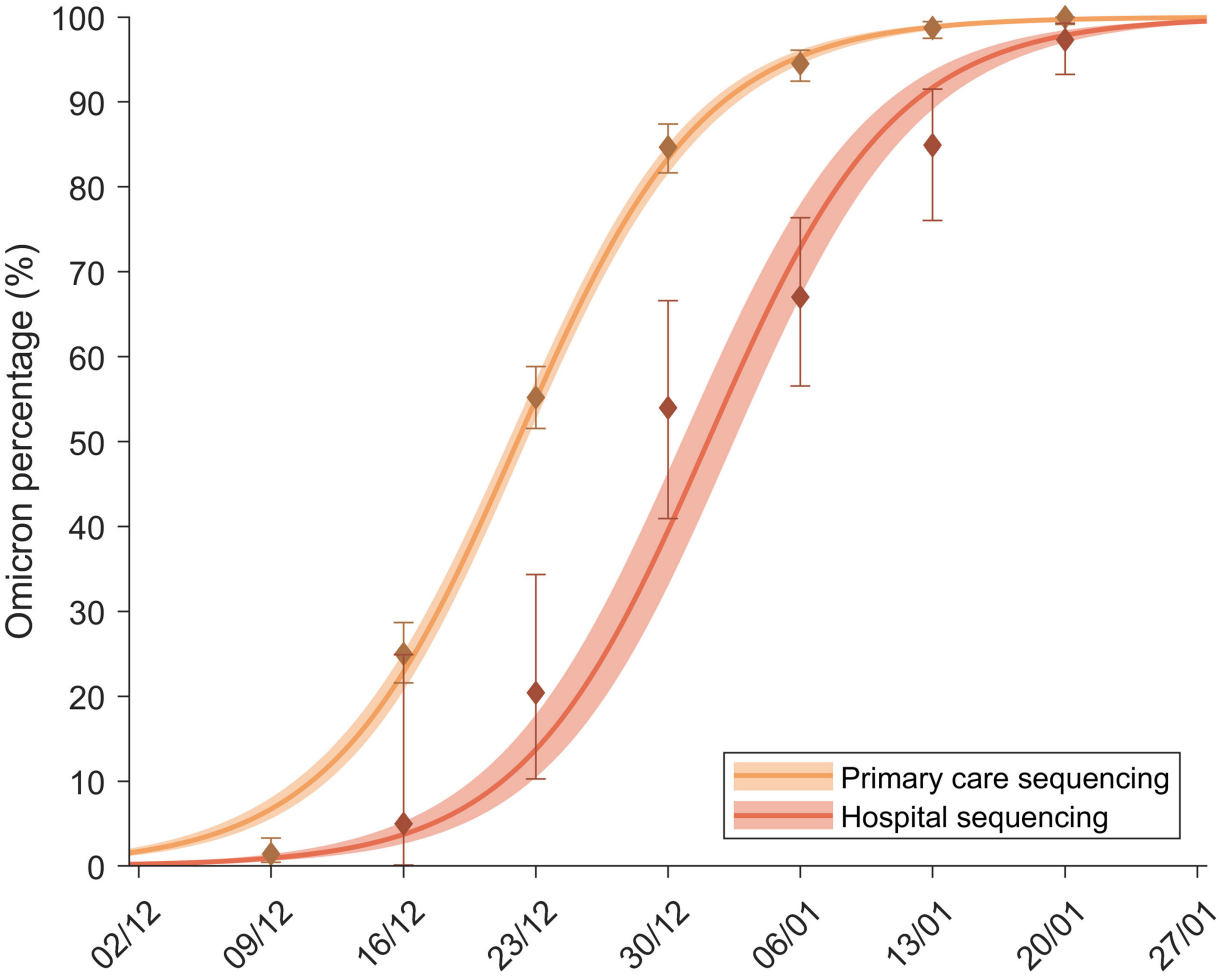
Percentage of omicron among screened samples of primary care patients (yellow) and hospital patients (orange). The continuous lines show the substitution model fitted to each dataset.

The fraction of samples from SARS-CoV-2 suggestive of the omicron variant based on SGTF in primary care and hospitals is shown in Figure 3. The substitution process was delayed in hospitals compared with primary care due to two factors. First, there is a delay between detection and admittance to hospitals because of the natural evolution of the disease that we have computed to be, on average, *T* = 4.75 [4.62, 4.88] days. Second, a reduction in the proportion of cases requiring admission also affected the delay between the two substitution curves, as discussed in Methodology. The substitution model fitted both substitution processes to obtain the RR for hospitalization. The obtained parameters from the fit values can be observed in Table 4. From this fit, the average hospitalization RR at the population level, α, was estimated between 0.19 and 0.50, a reduction in risk of between 80 and 50%.

**Table 4:** Values of the parameters obtained from the fitting of the substitution model to primary care screened samples and to the joint fitting of primary care and hospital variants data.

**Parameter**	**Definition**	**Value**	**95% CI**
**Only primary care model**			
ξ_*c*_	Fit of the ratio between initial cases of both variants	0.0648	[0.0510, 0.0785]
Δβ	Fit of the differences in exponential functions	0.209	[0.196, 0.222]
**Primary care** **+** **Hospitalizations model**			
ξ_*c*_	Fit of the ratio between initial cases of both variants	0.0721	[0.0579, 0.0863]
ξ_*H*_	Fit of the ratio between initial hospitalizations of both variants	0.00941	[0.00586, 0.01298]
Δβ	Fit of the differences in exponential functions	0.202	[0.190, 0.214]
α	Fit of the rate ratios of hospitalizations between both variants	0.341	[0.185, 0.496]

## Discussion

Ecological analysis of the percentage of hospitalization and ICU admissions in different subgroups of detected SARS-CoV-2 cases points to a systematic drop in the severity of the disease caused by the omicron variant with respect to the delta variant. The RR of hospitalization and ICUs is lower than 1 for all age cohorts and is also observed independently of vaccination status. For non-vaccinated, fully vaccinated, and boosted groups, there is a reduction in the percentage of detected cases requiring hospitalization or ICU care. Most age groups and vaccination status groups have a reduction in hospitalization of 40–80% and in ICU admission of 60–90%. Such a systematic reduction regardless of age and vaccination status suggests an overall reduction in severity which could be intrinsic to the omicron variant, and not due merely to the high level of immunization of the population. The fact is that the RR in ICU admission is systematically smaller than in hospitalization points in the same direction.

Our study found an overall 54% and 75% reduced risk of hospitalization and ICU admission for omicron cases compared to delta, respectively. While our ecological analysis has the known limitation of making direct comparison between subgroups unreliable, the general average results are consistent with those observed in other studies. For instance, in Norway, researchers found that omicron was associated with a 73% reduced risk of hospitalization (4). National reports from the United Kingdom observed 67% reduced hospital admission (hazard ratio 0.33, 95% CI: 0.30 to 0.37) with omicron (10), while an article also from the UK found that the risk of severe outcomes following SARS-CoV-2 infection was lower with omicron compared to delta [HR of 0.56 (95% CI: 0.54–0.58) for hospital attendances and 0.41 (95% CI: 0.39–0.43) and 0.31 (95% CI: 0.26–0.37) for hospital admission and death], with significant age variations (3). In addition, in the United States, a study estimated reductions of hospital and ICU admissions for omicron compared to delta of 38 and 41%, respectively—lower than those from our study (11). Finally, in Canada, a study found that the risk of hospitalization and death was 59% lower (HR = 0.41; 95% CI: 0.30–0.55) among omicron cases compared to delta cases, while the risk of UCI admission or death was 81% lower (HR = 0.19, 95% CI: 0.09–0.39) (5). All these reductions were observed for both unvaccinated and vaccinated individuals, as in our study (3, 4, 11), suggesting a reduction in the intrinsic severity of the omicron variant.

We stress here that our analysis does not include reinfections. We only study patients that are first cases in the data system. We do not have information on antibody status for these patients, so we cannot be sure that all patients did have the disease for the first time, since, during the first wave a part of the cases were undetected. After the first wave, contact tracing protocols were set-up and asymptomatic cases typically represented 50% of the cases detected according to our health records. Nevertheless, even if some cases were hidden reinfections, the fact is that the reinfections that were detected (and disregarded in this analysis) during the period under analysis were very low (below 8%). In this sense, it is highly unlikely that a possible highly asymmetric hidden reinfection with omicron vs. delta can explain a systematic 75% reduction in ICU admissions with constant admission protocols, pointing again toward omicron being an inherently less severe variant than delta.

Similarly, as the periods used to analyze the RR in hospitalization and ICU are relatively short, other environmental effects that could lead to a reduction in severity, such as a better immunization system or weather, are highly unlikely. In this sense, we performed a substitution analysis that investigates a global averaged reduction in the RR tracking information in a continuous way. Analysis of the substitution process in primary care settings and hospitalization led to similar increases in transmissibility. In that short period of time, the inferred RR in hospitalization is consistent with those observed ecologically before and after the substitution of delta by omicron. In other words, with an effective RR of around 0.34, the substitution analysis leads to an RR that is similar to those observed in the different subgroups in the ecological analysis.

The substitution model has the limitation of considering the same time frame for both variants. Despite this assumption, this model has been successfully applied to the substitution process of alpha and delta variants in the past (12). It provided estimates of an increase in transmissibility of around 100−200%, in line with but slightly lower than the estimation of Abbot et al. (13).

An important feature that did change during the period of the study is the number of people with a booster dose in the general population. The booster dose was available only to a reduced number of high-risk groups in September 2021. From November 2021, it was made available for the whole population at different stages depending on age and previous vaccine platform. The actual peak of third-dose administration happened just before Christmas in the Catalan Health System, the 18^th^ of December 2021, and its administration remained very high up until mid-January. The number of individuals aged >10 with two doses increased only from 82%, at the beginning of the study period (01 November 2021), to 85%, at the end (25 January 2022), mainly due to an increase in children coverage. On the contrary, the increase in booster coverage in the Catalan population older than 10 years was 2% at the beginning and 45% at the end of the study period. This leads to a low level of boosted individuals for the delta period (596) that represents a similar percentage (1.2%) of the overall number of cases detected during the delta period. This indicates the necessity to divide the cohorts in different vaccination statuses to check that all of them show a reduction in severity, but it is a limitation in the purely substitution model since the average hospitalization can be affected by the protection of the third dose.

Regarding whether the decrease in severity compensated for the increase in transmission in terms of the resources needed to face the epidemics, we observed that ICU admissions did not grow as a consequence of the substitution process and were thus compensated for, but did not compensate for general hospital admissions, as they did continue to grow. We must stress that this result is dependent on the particularities of the Catalan situation. First, it depended, necessarily, on the level of vaccination and population immunity since both strongly affect the level of protection against severe disease for a given number of cases (14–16). In this sense, it depends, as mentioned, on the particulars of the booster administration that became widespread precisely during January, in association with the decrease in transmission and hospitalization occupation. Second, our analysis cannot qualify severity according to WHO Clinical Progression Ordinal Scale and the associated resources that the omicron cases entail within the hospital compared to delta (17). A higher number of admissions might not mean a more complex picture in the hospital if the cases under observation are less severe and require less clinical support.

Finally, an important question arises regarding how useful this information may be in preparing health services for future waves. In this sense, we must stress that the ratios we present here are highly dependent on the particularities of the Catalan Health Care System. In other countries and areas, the ratio of hospitalization and ICU admission could be very different due to different diagnostic abilities (18), different guidelines for hospitalization vs. in-house control (19–21), and the specific definition of ICU, which in Catalonia is not limited to those patients with mechanical ventilation but also appropriate for patients requiring, or likely to require, advanced respiratory support with different oxygen therapies when saturation is <90%, and also included patients with chronic impairment of one or more organ systems who also require support for an acute reversible failure of another organ. Furthermore, the effort in detection is markedly different depending on the country/region, affecting the observed ratios.

Our study has important strengths. The large sample of first detected infections used in the study plus the fact that two very different methodologies provided comparable results regarding the average reduction in severity lends a significant robustness to our main result. Furthermore, the ratios obtained here can be used as general guidelines in the future as long as the omicron variant is dominant. They can also be critically compared and used in other countries. Continuously tracking these ratios and analyzing their deviations from the data presented here could be very useful, and they could be interpreted as warning signs if the ratios were to increase above the general baseline observed in this study.

## Data availability statement

The datasets regarding cases with its vaccination status, age cohort and sex are publicly available in https://analisi.transparenciacatalunya.cat/browse?q=covid&sortBy=relevance and https://dadescovid.cat/descarregues. Hospital lab information on variants is all included in the tables.

## Ethics statement

The studies involving human participants were reviewed and approved by CEIC Hospital Germans Tries i Pujol, Badalona, Spain (Project Code PI-22-131) and the Hospital Universitari Vall d'Hebron Clinical Research Ethics Committee (2022-533), Barcelona, Spain. Written informed consent for participation was not required for this study in accordance with the national legislation and the institutional requirements.

## Author contributions

MC: conceptualization, formal analysis, visualization, methodology, and software. EC: data curation, conceptualization, methodology, software, and writing—original draft. SA: conceptualization, formal analysis, methodology, and writing—original draft. CA: methodology, data curation, investigation, and writing—original draft. AA: methodology, data curation, and investigation. IB, AB, and VS: data curation and investigation. P-JC: data curation and validation. FF and MM: data curation, methodology, and formal analysis. EM: methodology and investigation. NM: formal analysis, methodology, and investigation. CP: conceptualization, data curation, formal analysis, supervision, and funding acquisition. DP-A: conceptualization, supervision, and writing—original draft. EA-L: conceptualization, formal analysis, supervision, project administration, and writing—original draft. All authors critically reviewed and edited the draft and approved the final draft.

## Funding

MC received funding from la Caixa Foundation ID 100010434, under agreement LCF/PR/GN17/50300003. MC, CP, and SA received funding from Ministerio de Ciencia, Innovación y Universidades and FEDER, with the project PGC2018-095456-B-I00.

## Conflict of interest

EA-L, CP, and SA report a relationship with Janssen-Cilag SA that includes consulting or advisory. DP-A reports a relationship with Amgen Inc that includes consulting or advisory, funding grants, and speaking and lecture fees. DP-A reports a relationship with Chesi-Taylor that includes funding grants. DP-A reports a relationship with Novartis AG that includes funding grants. DP-A reports a relationship with UCB Biopharma that includes consulting or advisory, funding grants, and speaking and lecture fees. DP-A reports a relationship with Astellas Pharma Inc that includes consulting or advisory. DP-A reports a relationship with AstraZeneca that includes consulting or advisory. DP-A reports a relationship with Johnson and Johnson SA that includes consulting or advisory. Janssen, on behalf of IMI-funded EHDEN and EMIF consortiums, and Synapse Management Partners have supported training programs organized by co-author DP-A's department and open for external participants. The remaining authors declare that the research was conducted in the absence of any commercial or financial relationships that could be construed as a potential conflict of interest.

## Publisher's note

All claims expressed in this article are solely those of the authors and do not necessarily represent those of their affiliated organizations, or those of the publisher, the editors and the reviewers. Any product that may be evaluated in this article, or claim that may be made by its manufacturer, is not guaranteed or endorsed by the publisher.
