# Corrigendum: Transmissibility, hospitalization, and intensive care admissions due to omicron compared to delta variants of SARS-CoV-2 in Catalonia: A cohort study and ecological analysis

**DOI:** 10.3389/fpubh.2022.1060328

**Published:** 2022-11-03

**Authors:** Martí Català, Ermengol Coma, Sergio Alonso, Cristina Andrés, Ignacio Blanco, Andrés Antón, Antoni E. Bordoy, Pere-Joan Cardona, Francesc Fina, Elisa Martró, Manuel Medina, Núria Mora, Verónica Saludes, Clara Prats, Daniel Prieto-Alhambra, Enrique Alvarez-Lacalle

**Affiliations:** ^1^Nuffield Department of Orthopedics, Rheumatology and Musculoskeletal Sciences (NDORMS), University of Oxford, Oxford, United Kingdom; ^2^Primary Care Services Information System (SISAP), Institut Català de la Salut (ICS), Barcelona, Spain; ^3^Physics Department, Universitat Politècnica de Catalunya, Barcelona, Spain; ^4^Respiratory Viruses Unit, Virology Section, Microbiology Department, Vall d'Hebron Hospital Universitari, Vall d'Hebron Institut de Recerca (VHIR), Vall d'Hebron Barcelona Hospital Campus, Barcelona, Spain; ^5^Biomedical Research Networking Center in Infectious Diseases (CIBERINF), Instituto de Salud Carlos III, Madrid, Spain; ^6^Clinical Genetics Department, Laboratori Clínic Metropolitana Nord, Hospital Universitari Germans Trias i Pujol, Institut d'Investigació en Ciències de la Salut Germans Trias i Pujol (IGTP), Badalona, Spain; ^7^Microbiology Department, Laboratori Clínic Metropolitana Nord, Hospital Universitari Germans Trias i Pujol, Institut d'Investigació en Ciències de la Salut Germans Trias i Pujol (IGTP), Badalona, Spain; ^8^Biomedical Research Networking Center in Respiratory Diseases (CIBERES), Instituto de Salud Carlos III, Madrid, Spain; ^9^Department of Genetics and Microbiology, Universitat Autònoma de Barcelona, Cerdanyola, Spain; ^10^Biomedical Research Networking Center in Epidemiology and Public Health (CIBERESP), Instituto de Salud Carlos III, Madrid, Spain

**Keywords:** COVID-19, severity, ecological study, cohorts, substitution model, severity and vaccination status

In the published article, there were errors in affiliations 5–10.

In affiliation 5, instead of “Biomedical Research Networking Center in Infectious Diseases CIBERINF, Instituto de Salud Carlos III, Madrid, Spain,” it should be “Biomedical Research Networking Center in Infectious Diseases (CIBERINF), Instituto de Salud Carlos III, Madrid, Spain.”

In affiliation 6, instead of “Clinical Genetics Department, Laboratori Clínic Metropolitana Nord, Hospital Universitari Germans Trias i Pujol, Institut Universitari Germans Trias i Pujol (IGTP), Badalona, Spain,” it should be “Clinical Genetics Department, Laboratori Clínic Metropolitana Nord, Hospital Universitari Germans Trias i Pujol, Institut d'Investigació en Ciències de la Salut Germans Trias i Pujol (IGTP), Badalona, Spain.”

In affiliation 7, instead of “Biomedical Research Networking Center in Infectious Diseases CIBERINF, Instituto de Salud Carlos III, Madrid, Spain,” it should be “Microbiology Department, Laboratori Clínic Metropolitana Nord, Hospital Universitari Germans Trias i Pujol, Institut d'Investigació en Ciències de la Salut Germans Trias i Pujol (IGTP), Badalona, Spain.”

In affiliation 8, instead of “Microbiology Department, Laboratori Clínic Metropolitana Nord, Hospital Universitari Germans Trias i Pujol, Institut Universitari Germans Trias i Pujol (IGTP), Badalona, Spain,” it should be “Biomedical Research Networking Center in Respiratory Diseases (CIBERES), Instituto de Salud Carlos III, Madrid, Spain.”

In affiliation 9, instead of “Biomedical Research Networking Center in Respiratory Diseases CIBERES, Instituto de Salud Carlos III, Madrid, Spain,” it should be “Department of Genetics and Microbiology, Universitat Autònoma de Barcelona, Cerdanyola, Spain.”

In affiliation 10, instead of “Department of Genetics and Microbiology, Universitat Autònoma de Barcelona, Cerdanyola, Spain,” it should be “Biomedical Research Networking Center in Epidemiology and Public Health (CIBERESP), Instituto de Salud Carlos III, Madrid, Spain.”

The authors apologize for these errors and state that this does not change the scientific conclusions of the article in any way. The original article has been updated.

## Publisher's note

All claims expressed in this article are solely those of the authors and do not necessarily represent those of their affiliated organizations, or those of the publisher, the editors and the reviewers. Any product that may be evaluated in this article, or claim that may be made by its manufacturer, is not guaranteed or endorsed by the publisher.

